# Retrospective study of elderly frequent attenders presenting with chest pain at emergency department

**DOI:** 10.1186/s12245-014-0035-2

**Published:** 2014-09-12

**Authors:** Faraz Zarisfi, Qi En Hong, Pauline See Joon Seah, Huihua Li, Susan Yap, Marcus Eng Hock Ong

**Affiliations:** 1 Department of Emergency Medicine, Singapore General Hospital, Outram Road, Singapore 169608, Singapore; 2 Yong Loo Lin School of Medicine, National University of Singapore, 1E Kent Ridge Road, Singapore 119228, Singapore; 3 Health Services Research Unit, Division of Research, Singapore General Hospital, 226 Outram Road Blk A Level 2, Singapore 169039, Singapore

**Keywords:** Chest pain, Frequent attenders, Elderly, Emergency department

## Abstract

**Background:**

The aims of the study were to identify the characteristics of elderly frequent attenders to the emergency department (ED) presenting with chest pain and to assess the 1-year prognosis for developing adverse cardiac events.

**Findings:**

Patients over 75 years old, with four or more attendances to the ED between 1 January 2010 and 31 December 2010 with at least one attendance due to chest pain, were selected from a database. Data was collected on demographic details, visit history, disposition and admission outcomes. Each patient was followed up for 12 months after the index episode via the hospital electronic registry for adverse cardiac outcome. Adverse cardiac outcomes included death from cardiac event, acute myocardial infarction (ST elevation myocardial infarction (STEMI)/non-ST elevation myocardial infarction (NSTEMI)) or unstable angina. A total of 158 patients with 4 or more visits to the ED accounted for 290 visits with chest pain during 2010. There is a high prevalence of coronary risk factors in this cohort (hypertension 92.4%, hyperlipidaemia 65.2%, diabetes 49.4% and smoking 26.6%). The hospital admission rate was also high at 83.5%. Over the ensuing 12 months, 8 patients died of a primary cardiac event and a further 29 patients developed 36 non-fatal cardiac events. We could not establish any significant relationship between increase in adverse cardiac outcome and individual risk factors or even two or more risk factors (*P* = 0.0572). Patients with two or more attendances with chest pain were more likely to develop adverse cardiac outcome (*P* = 0.0068).

**Conclusions:**

Elderly frequent attenders to the ED, who present with chest pain, have more cardiac risk factors and are more likely to develop adverse coronary outcomes if they re-attend with chest pain.

## 1 Findings

### 1.1 Introduction

In keeping with the rest of the industrialised and developed world, there has been a changing demographic in Singapore [1],[2]. There has been a steadily increasing age of patients attending emergency departments and a steadily increasing frequency of attendance. The Department of Emergency Medicine at Singapore General Hospital is an adult emergency department (ED) in a tertiary care hospital, housing the National Heart Centre in Singapore; the department has roughly 150,000 attendances per annum.

Although most of the available literature on frequent attenders is concerned with patients with mental health and social issues, qualitative studies of staff attitudes suggest an assumption of ‘low risk’ in this patient group, by healthcare staff [3]-[7]. We have observed within our frequent attenders a significant proportion of elderly patients (age > 75). This is in keeping with other studies carried out at other institutions in Singapore [8].

Chest pain is a common presenting complaint for ED attendance; in our department, this accounts for approximately 7% of our attendances. The risk of missing ongoing acute coronary syndrome has been a well-documented and researched issue [9]-[11]. Our concern was whether in an elderly population of frequent attenders the risk factors and outcomes would be altered in any way.

The aim of this study was to review this specific group of patients in an effort to identify their characteristics and associated risk factors.

## 2 Methods

**Figure 1:**
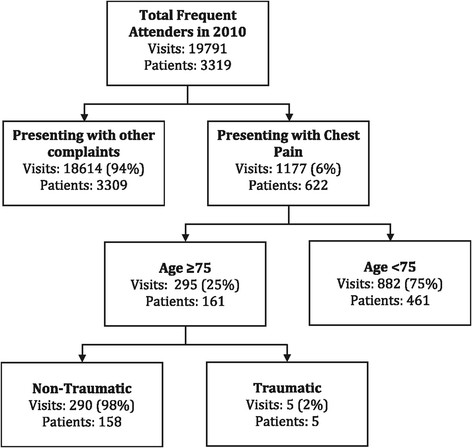
Selection process of patient cohort.

Patients with more than four attendances in 1 year were deemed as regular attenders [12]. The departmental electronic registry system was interrogated. Patients aged 75 and older attending the department four or more times between 1 January 2010 and 31 December 2010, where at least one visit was due to non-traumatic chest pain, were identified (Figure 1). An institutional review board approval was obtained (CIRB/2011/037/C).

Data on demographic and bio-data variables, visit details, disposition and admission outcomes were collected from the ED computerised notes (EMERGE). Each patient was followed up for 12 months via hospital electronic registry (Citrix Systems) from the index visit with chest pain to assess for adverse cardiac outcomes. Adverse cardiac outcomes were defined as death where the cause of death is documented as a primary cardiac event and further episodes of acute coronary syndrome (ST elevation myocardial infarction (STEMI), non-ST elevation myocardial infarction (NSTEMI) or unstable angina). Confirmation of adverse outcome was vetted by reviewing clinicians. This was achieved by reviewing ED and ward notes and discharge diagnoses, supported by ECG, cardiac enzymes, angiography and perfusion scans to differentiate STEMI, NSTEMI and unstable angina. Study data were collected and managed using Research Electronic Data Capture (REDCap) electronic data capture tools hosted at Singapore General Hospital.

Statistical analysis was performed using SPSS 17.0 (SPSS Inc., Chicago, IL, USA). Statistical significance of the data was assessed by performing two-tailed *χ*^2^ analysis.

## 3 Results

Over the 1-year period in 2010, 3,319 patients accounted for 19,791 ED visits and were highlighted as recurrent attenders. Within this group, we identified 290 attendances for non-traumatic chest pain to the ED, accounted for by 158 patients over the age of 75 (Figure 1). These patients are 57.6% female and 80.4% of Chinese background. The majority (91.4%) of these patients self-presented to the ED with a small minority of patients (8.6%) being brought by ambulance. Seasonal, daily and diurnal variations in attendance followed the regular pattern of attendance for our emergency department.

The prevalence of cardiac risk factors was high in our cohort (Table 1). Over 90% of the cohort have hypertension, underlying coronary artery disease is common, roughly half of the cohort has diabetes and over one quarter were still smoking at index attendance episode. Data for family history was not fully available; it is therefore not included in subsequent calculations. There were no risk-free patients in our cohort. The coded primary diagnosis for each index episode is shown in Table 2.

**Table 1:** Prevalence of cardiac risk factors

**Risk factor**	**Percentage (*n*)**
Hypertension	92.4 (146)
Known CAD	85.4 (135)
Hyperlipidaemia	65.2 (103)
Diabetes	49.4 (78)
Smoking	26.6 (42)

**Table 2:** Diagnosis at primary attendance

**Primary diagnosis**	**Percentage**
Acute myocardial infarct	8.3
Acute ischaemic heart disease	26.9
Cardiac failure	0.6
Non-cardiac chest pain	1.4
Chest pain, not otherwise specified	62.8

Over three quarters (78.3%) of the cohort were admitted directly. Of the 11.4% in the ED observation unit, almost half were admitted for further workup or due to unresolved symptoms; 6.9% were discharged directly from the ED and 3.4% self-discharged from the department or ward. The total hospital admission rate was 83.5%. Of the total ward admissions, 98% were discharged alive, with a median length of stay of 3 days (range 0 to 27); 1.2% died in the hospital and 0.8% self-discharged (Table 3).

**Table 3:** Disposition at primary attendance

**Disposition**	**Percentage**
Direct ward admission	78.3
DEM observation unit	11.4
Admitted	5.2
Discharged	6.2
Direct discharge	6.9
Self-discharge	3.4

Adverse cardiac outcome was defined as death from a cardiac cause or a non-fatal acute coronary syndrome (STEMI, NSTEMI, unstable angina) within 1 year of the index episode with chest pain. There were 19 deaths in the group over a 1-year period (12% 1-year mortality); of these, 8 were attributed to a primary cardiac cause. Over the year, a further 29 patients in the surviving group developed 36 non-fatal ACS episodes. Twenty-five episodes occurred in the first 6 months after the initial episode of chest pain and 11 in the subsequent 6 months.

Statistical analysis of adverse cardiac outcome against demographic and cardiac risk factors (Table 4) shows no association with gender or ethnicity, no individual risk factor was associated with adverse cardiac outcome and the presence of two or more risk factors did not show a statistically significant increase in risk (*P* = 0.0572). In our cohort, multiple attenders presenting with a single episode of chest pain had a lower rate of adverse cardiac outcome than those with multiple chest pain attendance (Table 5).

**Table 4:** Analysis of adverse cardiac outcome against demographic and cardiac risk factors

	**Adverse cardiac outcome**				***P*** **value**
	**Negative (*n*** **= 121)**		**Positive (*n*** **= 37)**		
Gender					
Male	51		16		0.45
Female	70		21		
Ethnicity					
Chinese	100	82.6% (0.75 to 0.88)	27	73% (0.57 to 0.85)	
Malay	7	5.8% (0.03 to 0.12)	6	16.2% (0.07 to 0.32)	
Indian	10	8.3% (0.04 to 0.15)	4	10.8% (0.04 to 0.25)	
Other	4	3.3% (0.01 to 0.08)	0	0% (0 to 0.11)	
Risk factors					
Hypertension	110	90.9% (0.84 to 0.95)	36	97.3% (0.85 to 0.99)	
CAD	101	83.5% (0.76 to 0.89)	34	91.9% (0.78 to 0.98)	
Hyperlipidemia	76	62.8% (0.54 to 0.71)	27	73.0% (0.57 to 0.85)	
Diabetes	57	47.1% (0.38 to 0.56)	21	56.8% (0.41 to 0.71)	
Smoking	34	28.1% (0.21 to 0.37)	8	21.6% (0.11 to 0.37)	
≥2 risk factors	110	90.9% (0.84 to 0.95)	37	100% (0.88 to 1)	0.0572

CAD, coronary artery disease. Confidence intervals are in parentheses.

**Table 5:** Incidence of adverse coronary outcome in the group

**Frequency of attendance**	**Number**	**Frequency of adverse outcome**
1	102	17 (16.7%)
2+	56	20 (35.7%)

*P* = 0.0068.

## 4 Discussion

There is a high prevalence of cardiac risk factors in our cohort population. Comparison with pooled international data from patients with ischaemic coronary heart disease enrolled in different trial registries [13] showed far lower prevalence rates for an age-matched population. Hypertension 51.3% was compared with our cohort of 92.4%, dyslipidaemia 24.6% compared with 65.2%, diabetes 20.1% compared with 49.4% and smoking 9.9% compared with 26.6% in our study group. The high prevalence of risk factors is reflected in the high admission rate. Of our cohort, 29/158 (18.4%) had an adverse cardiac outcome within 1 year and were thus positive for the primary outcome measure. This is difficult to compare as we could not identify any other studies with our cohort of patients elsewhere in the literature.

Repeat attendance with chest pain in multiple attenders over the age of 75 is associated with an increase in 1-year adverse cardiac outcome rate. The assumption that recurrent attenders to the emergency department do not necessarily have serious pathology must be challenged in this specific scenario.

There are limitations to this study; our sample size is small, and this represents an underpowered statistic. We could not show if having two or more cardiac risk factors would be associated with an increased risk of cardiac event within 1 year of chest pain presentation in this group. This would have been in keeping with the work of Khot and colleagues [13] who found a reduction in the age at which the index CHD event occurred with multiplicity of risk factors.

In most studies and guidelines, 65 is used as the upper age limit in keeping with TIMI data, whilst our choice of 75 due to the cohort definition of elderly recurrent attenders does not alter the quality of our data; it would have allowed our result comparison and reference to international and regional studies [14]. Our data was retrospectively collected and analysed, leading to bias. Although the study impact is limited to a small subgroup of total emergency department workload, we have nonetheless demonstrated a high-risk population.

## 5 Conclusions

We found in patients over the age of 75 that those with two or more attendances with chest pain out of four or more total attendances were more likely to develop adverse cardiac events. Whether the frequency of attendance or exclusivity of chest pain attendance is responsible for this effect is a question that will need further study.

## Abbreviations

ED: emergency department

NSTEMI: non-ST elevation myocardial infarction

STEMI: ST elevation myocardial infarction

## Competing interests

The authors declare that they have no competing interests.

## Authors’ contributions

QEH, FZ and MEHO conceived and designed the study. QEH and SY collected the data. QEH, FZ and HL analysed the data. FZ, QEH, MEHO and SY drafted the manuscript. All authors read and approved the final manuscript.
